# Proteinuria Assessment and Therapeutic Implementation in Chronic Kidney Disease Patients—A Clinical Audit on KDIGO (“Kidney Disease: Improving Global Outcomes”) Guidelines

**DOI:** 10.3390/jcm13175335

**Published:** 2024-09-09

**Authors:** Gabriela Adelakun, Maria Boesing, Munachimso Kizito Mbata, Zahra Pasha, Giorgia Lüthi-Corridori, Fabienne Jaun, Felix Burkhalter, Jörg D. Leuppi

**Affiliations:** 1 Institute of Internal Medicine, Cantonal Hospital Baselland, Mühlemattstrasse 24, 4410 Liestal, Switzerland; 2 Medical Faculty, University of Basel, Klingelbergstrasse 61, 4056 Basel, Switzerland; 3 Department of Clinical Nephrology, Cantonal Hospital of Baselland, Rheinstrasse 26, 4410 Liestal, Switzerland

**Keywords:** CKD, proteinuria, albuminuria, RASi, KDIGO guidelines, audit

## Abstract

**Background/Objectives**: Chronic kidney disease (CKD) is a major health problem with a rising prevalence due to comorbidities like diabetes and hypertension. The aim of this research was to audit the assessment and therapeutic management of proteinuria in CKD patients at the Cantonal Hospital Baselland (KSBL) in Switzerland and determine associations between patient comorbidities, rehospitalisation, death, and the quality of therapeutic management. **Methods**: We analysed data from 427 adults with CKD (eGFR < 45 mL/min/1.73 m^2^) hospitalised on the internal medicine ward in 2022. **Results**: The mean age was 85 years (range: 79–89), 45.9% were female, and the median eGFR was 32.8 mL/min/1.73 m^2^ (range: 25–40). Proteinuria assessment was performed in 120 (28.1%) patients (the ProtU group), and a corresponding treatment was prescribed in 59%. The ProtU group had a higher quota of patients with diabetes (44.1% vs. 33%, *p* = 0.048) and obesity (21.2% vs. 12.5%, *p* = 0.039) when compared to the group without proteinuria assessment (the Ustix group). Twelve-month survival was not significantly different between the groups (HR: 0.75; 95% CI: 0.488–1.154; *p*-value = 0.191). However, survival was significantly better in patients who received an antiproteinuric treatment compared to those who did not (HR: 0.30; 95% CI: 0.121–0.0761; *p* = 0.011). **Conclusions**: Improvements need to be made in managing CKD at the KSBL in accordance with the guidelines.

## 1. Introduction

Chronic kidney disease (CKD) is defined by a sustained decline in the estimated glomerular filtration rate (eGFR) to less than 60 mL/min/1.73 m^2^ or kidney damage for over three months irrespective of the cause [1,2]. It is classified by considering the cause, eGFR, and albuminuria category [1,3]. The prevalence of CKD is still increasing worldwide, mostly due to comorbidities like diabetes and hypertension, which are the two most frequent causes, and the ageing population (>60 years) [3,4,5].

In 2017, the global prevalence of CKD was 9.1%; currently, over 10% of the global population is affected by CKD, a condition associated with high morbidity and mortality [3,6]. In 2016, a systematic review and meta-analysis determined that the global mean of CKD prevalence was around 13.4%, with the majority being category G3, at a prevalence of 7.6% [5]. Between 1990 and 2017, CKD was responsible for 4.6% of all-age mortality worldwide [7]. A study organised by the Global Burden of Disease (GBD) Chronic Kidney Disease Collaboration has forecasted that CKD will rank up to the fifth leading cause of global mortality by 2040 [7]. Another study by Forni et al. on the prevalence of CKD in the Swiss population showed that 1 in 10 adults is affected by renal failure, corresponding to around 10.4% of adults [4]. Consequently, CKD causes a significant burden on healthcare systems and negatively affects the quality of life of affected individuals [3,5,8].

It is known that CKD is associated with other chronic diseases, such as cardiovascular diseases, diabetes, and obesity, as well as tobacco abuse [2]. In the early stages of CKD, subjects are usually asymptomatic. As it progresses, it leads to complications such as acidosis, hyperuricemia, mineral and bone disorders, anaemia, hyperparathyroidism, and others, which is why adequate and regular assessment is important [2,9,10]. If preventive and therapeutic measures are lacking, CKD progresses to end-stage kidney disease (ESKD), necessitating renal replacement therapy such as dialysis or kidney transplantation. One major determinant for ESKD is proteinuria [11]. Proteinuria is a general term for different types of proteins found in urine including albuminuria [12].

In 2012, clinical practice guidelines were developed for the management of CKD by the “Kidney Disease: Improving Global Outcomes” (KDIGO) organisation [1]. These guidelines state that albuminuria is an “independent predictor of important clinical outcomes including CKD progression, ESKD, acute kidney injury, cardiovascular mortality, and all-cause mortality” [1]. Hence, the addition of the albuminuria category to the KDIGO 2012 classification system reinforces the significance of the detection of albuminuria. Calculation of the urinary protein-to-creatinine ratio (UPCR), or preferably albumin-to-creatinine ratio (ACR) [10], in a random urine sample can be used to quantify protein loss [1,13,14,15]. In physiological conditions, every human excretes proteins in their urine; the proteins usually found are albumin, immunoglobulins, and Tamm–Horsfall proteins, with a UPCR < 20 mg/mmol (0.2 g/24 h) or an ACR < 3 mg/mmol being considered physiological [16,17]. Proteinuria can be transient or orthostatic; in these cases, it is benign and does not need to be treated [16].

Pathophysiologically, proteinuria results from a variety of conditions, including (1) glomerular lesions (glomerulopathy), where it is primarily composed of albumin; (2) a deficit of reabsorption by the tubular cells, in which there are mainly low-molecular-weight proteins (for example, Fanconi syndrome); (3) an overflow of light chains (Bence Jones proteinuria); and (4) postrenal proteinuria (inflammation of the urinary tract). Glomerulopathies are the most frequent causes [16,18,19]. Elevated values of proteinuria are a marker for kidney damage and should be regularly assessed [20]. A treatment should be initiated to prevent adverse outcomes [2]. Angiotensin-converting enzyme inhibitors (ACEis) and angiotensin receptor blockers (ARBs) have proven to slow progression from mild or moderate CKD stages, thus delaying the occurrence of ESKD [21,22,23]. ARBs and ACEis inhibit glomerular hyperfiltration, consequently preventing a decline in the eGFR or an increase in proteinuria [24,25]. In one study, a 26% decrease in proteinuria was noted within 3 months in patients given losartan, with a further decrease of over 40% within 3 years, and a profound improvement in renal survival [20]. Nevertheless, these treatments should not be used as a combination therapy due to adverse outcomes and a lack of clear benefits [12]. Previous studies have shown a lack of proteinuria management in CKD patients, recognising a substantial need for improvement in this area to meet the guidelines [26,27,28,29]. Despite the importance of CKD and proteinuria management, an audit investigating guideline compliance has not yet been published in Switzerland.

The aim of this study was to audit the assessment and therapeutic management of proteinuria in CKD patients on the internal medicine ward at the Cantonal Hospital Baselland (KSBL) in Switzerland. Furthermore, we aimed to determine associations between the quality of proteinuria management and patient outcomes. This audit is a component of the QUA-DIT (Quality Evaluation of Hospital Care Through Audits) project, which aims to enhance hospital care by evaluating adherence to disease-specific clinical guidelines across key areas in internal medicine at the KSBL, ultimately seeking to improve patient outcomes through comprehensive quality-control programmes [30,31,32,33,34,35].

## 2. Materials and Methods

### 2.1. Study Design and Patient Selection

**Figure 1:**
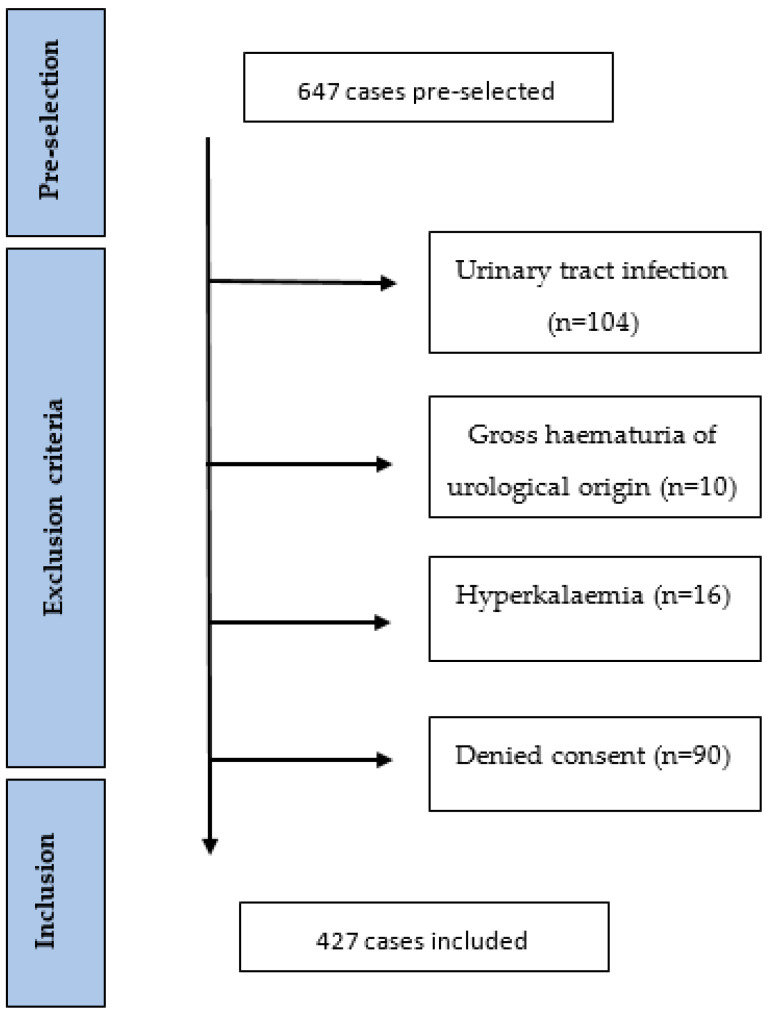
Flowchart diagram.

We conducted a retrospective, observational, single-centre study at the KSBL, a public teaching hospital. We studied files of adult patients with a diagnosis of CKD who were hospitalised on the internal medicine ward in 2022 in one of the KSBL sites (Bruderholz or Liestal) and had at least one eGFR value < 45 mL/min/1.73 m^2^ during their stay. The eGFR threshold of 45 mL/min/1.73 m^2^ (KDIGO G3b) was chosen because, according to the guidelines, proteinuria should be assessed at least twice a year in this CKD stage. Cases coded with one of the ICD-10 codes—N.18.3, N18.4, N18.5, N18.80, N18.89, or N18.9—were selected and consolidated with a list of cases fulfilling the eGFR criterion, resulting in 647 cases [36]. Patients with at least one of the following exclusion criteria were excluded from the analysis: urinary tract infection (UTI); gross haematuria of urological origin; hyperkalaemia; denial of general research consent. Patients with hyperkalaemia were excluded because we hypothesised that doctors may not want to initiate antiproteinuric treatment in patients who already have or are prone to hyperkalaemia. The KDIGO 2024 guidelines also state that reduction/discontinuation should be considered in patients with uncontrolled hyperkalaemia [37]. Figure 1 shows an overview of the included and excluded patients. Because this is an audit focusing on clinical processes, every eligible case was included into the analysis, even if one patient was hospitalised several times during the study period. Serum and urine creatinine, urine total protein, and albumin levels were determined in the Liestal laboratory using Cobas 8000 instruments from Roche Diagnostics in Liestal,,Switzerland (c502 module). Later, respective urine determination allowed us to calculate the ACR/PCR as a ratio. The eGFR was calculated according to the CKD-EPI formula [38] and patients were classified into CKD stages according to the KDIGO definition, as follows: category G3b (eGFR: 30–44 mL/min/1.73 m^2^); category G4 (eGFR: 15–29 mL/min/1.73 m^2^); category G5 (eGFR: <15 mL/min/1.73 m^2^) [1]. Evaluation of the urinalyses was carried out with Cobas 6500 from Roche Diagnostics in Liestal, Switzerland.

### 2.2. Data Collection and Management

The collection of clinical routine data was performed manually from the electronic patient files and entered into a Research Electronic Data Capture (RedCap^®^, version 13.8.1) database. RedCap^®^ is a platform designed to collect and analyse research data [39,40]. Patient files included discharge reports, patient history, nursing documentation, and laboratory records. We used the KDIGO 2012 guidelines as the state-of-the-art comparator [1]. The assessed guidelines are listed in Table 1.

### 2.3. Statistical Analyses

To evaluate the assessment and management of proteinuria, we divided the included patients into two groups: the “ProtU” group and the “Ustix” group. Patients in the ProtU group were subjected to a quantification of proteinuria and those in the Ustix group were not. Patients from the ProtU group were further classified regarding whether or not proteinuria was found, and whether or not a renin angiotensin system inhibitor (RASi) was indicated, and eventually prescribed. Statistical analyses were performed using RedCap^®^, R statistical software, version 4.0.3 and Microsoft Excel, version 16.0.5278.1000. Adherence to KDIGO guidelines was analysed in a descriptive manner. Continuous variables were shown using the mean +/− standard deviation (SD) when normally distributed, or the median and interquartile range (IQR) when not normally distributed. The following patient outcomes were compared between the groups: rehospitalisation within 6 months and death within 1 year.

To assess group-wise differences, the Mann–Whitney U test was used for continuous variables and the Chi Square test was used for categorical variables. “Time to event” data (i.e., time to rehospitalisation, time to death) were analysed with Cox-proportional hazards models, adjusted for sex, age, eGFR, and diabetes. We chose these variables as potential confounders because of an uneven distribution between the ProtU and Ustix groups, and based on expert knowledge of the potential effects of this on the respective outcomes. We confirmed the proportional hazards assumption using scaled Schoenfeld residuals with the function “cox.zph ()” in R version 4.0.3. Survival and rehospitalisation were visualised with Kaplan–Meier curves, generated with R version 4.0.3., and *p* < 0.05 was considered statistically significant.

### 2.4. Ethical Approval

This study was reviewed and approved by the Ethics Committee of Northwest and Central Switzerland (EKNZ; BASEC Project-ID 2023-01079).

## 3. Results

### 3.1. Baseline Characteristics

Of the 647 pre-selected cases, 220 were excluded (34%) based on the exclusion criteria. The remaining 427 cases were included in this audit (Figure 1). Table 2 presents the baseline characteristics of all 427 cases, divided by group according to whether or not a proteinuria assessment was performed. In the ProtU group, a UACR/UPCR measurement was performed; in the Ustix group, one was not. Overall, proteinuria was assessed in 28.1% of the cases (120/427). In the Ustix group, 176 (57.3%) underwent a urinalysis, of which 72 (40.9%) had traces of proteinuria. The mean age was 85 years, with a range of 79–89. Overall, 196 (45.9%) subjects were female. Moreover, 268 (62.8%), 123 (28.8%), and 36 (8.4%) patients were in the GFR categories of G3b, G4, and G5, respectively. In regard to comorbidities, 326 (77.4%) subjects had hypertension, 152 (36.1%) had diabetes, 63 (15%) had obesity, and 69 (16.4%) had a history of smoking or were active smokers. The proportions of patients with diabetes mellitus and obesity were significantly higher in the ProtU group than in the Ustix group (see Table 2).

### 3.2. Proteinuria Assessment

**Figure 2:**
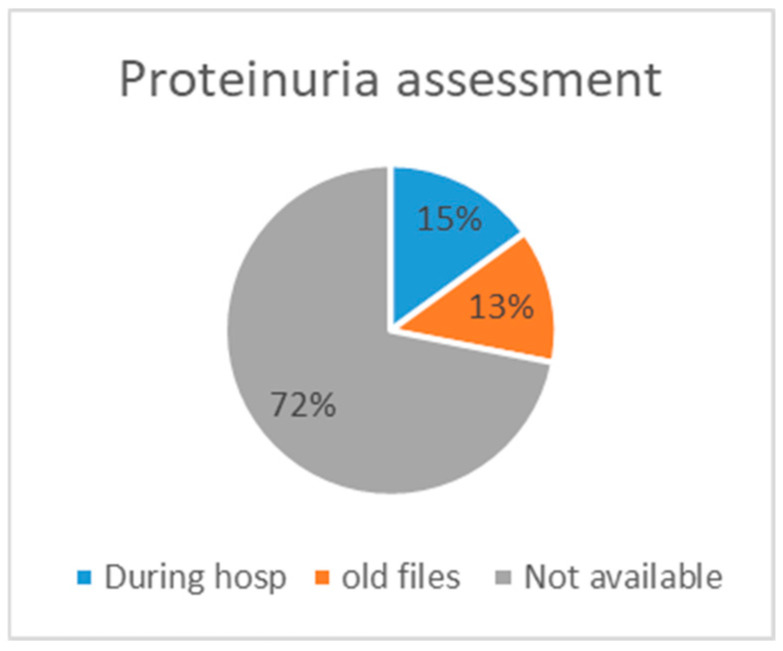
Assessment availability.

Sixty-four (15%) patients were assessed for proteinuria during their hospitalisation; for fifty-six (13%) cases, the proteinuria value was found in old files, and in 307 (72%) cases, there was no proteinuria value available (Figure 2). Table 3 presents the different CKD categories of patients whose proteinuria was assessed, as well as information on the implementation of treatment, the amount of referrals to a specialist, and the rates of rehospitalisation within 6 months and death within 1 year. The largest group was category G3bA1, with 51.6%; category G5A2 was the smallest, with 11.4%. However, when evaluating severe CKD (G5, eGFR 15–29), the severity of glomerular filtration seems proportional to the severity of albuminuria. Table 3 illustrates that in category G4, the largest proportion is represented by severe albuminuria (44.8%). The same applies to G5—the highest percentage exhibits severe albuminuria (31%). The same pattern is also apparent for terminal CKD and UPCR, at 45.2% in the G4 group and 22.6% in the G5 group. Additionally, patients with severe albuminuria were more prone to be referred to a nephrologist (37.9%) compared to those with normal to mild (9.7%) and moderate (11.4%) albuminuria. Rehospitalisation was more frequent in subjects with severe albuminuria (41.1%) compared to those with normal to mild (35.5%) and moderate albuminuria (31.8%) (Table 3). For better illustration, Table 4 represents the indication of RASi prescriptions and the number of cases in which they were prescribed. Most of these patients received an albuminuria assessment, and 21 cases with an indication for RASi had both UACR and UPCR assessments.

**Figure 3:**
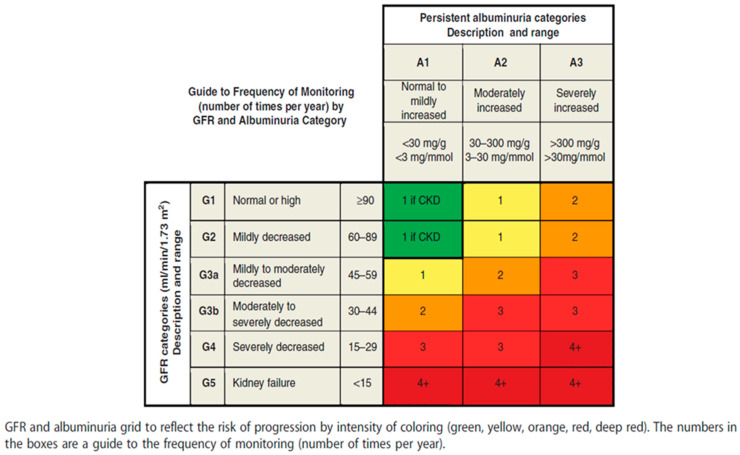
Frequency of monitoring by GFR value and albuminuria category (Inker, Astor et al., 2014 [1]—reprinted with permission).

According to the KDIGO 2012 guidelines, the albumin excretion rate or total protein excretion rate should be estimated at least twice a year from GFR category G3b onwards (see Figure 3) [1]. Furthermore, a RASi treatment should be initiated when albuminuria is at category A2 in diabetic patients and category A3 for non-diabetics, as well as UPCR ≥ 50 mg/mmol.

### 3.3. Proteinuria Treatment and Comorbidities

The prevalence of a pathological UACR or UPCR was 46.7%, corresponding to 56 out of 120 cases; thus, an antiproteinuric treatment was indicated in these cases. Of these, 45 (80.4%) had hypertension, 32 (57.1%) were diabetic, 12 (21.4%) were obese, and 11 (19.6%) were active smokers or had a history of smoking. Ultimately, only 33/56 (59%) cases were treated with a RASi. Of those for whom a RASi was indicated, 15 (51.7%) patients received an ARB or ACEi in the A3 group, and 20 (64.5%) received a corresponding treatment in the UPCR ≥ 50 mg/mmol group (Table 3). The KDIGO 2012 guidelines suggest a treatment for diabetics with A2 albuminuria (Table 1). In this study, 17 out of 44 (38.6%) individuals in the A2 group were diabetic patients—of which 12 (70.6%) were treated with a RASi.

**Figure 4:**
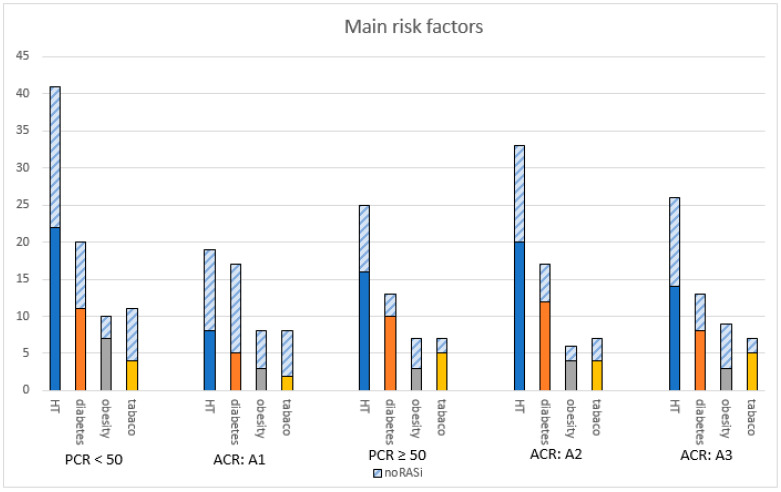
Patients with main risk factors in CKD. Abbreviations: ACR = albumin-to-creatinine ratio, HT = hypertension, PCR = protein-to-creatinine ratio, RASi = renin angiotensin system inhibitor.

Figure 4 shows the number of patients for the main comorbidities in CKD by proteinuria severity. In the group with mild proteinuria (A1) under RASi treatment, 8 out of 19 (42.1%) had high blood pressure (BP), 5 out of 17 (29.4%) were diabetic, 3 out of 8 (37.5%) were obese, and 2 out of 8 (25%) were active or former smokers. In the A3 group, regarding those who received a RASi, 14 out of 26 (53.8%) patients had hypertension, 8 out of 13 (61.5%) were diabetic, 5 out of 7 (71.4%) were former or active smokers, and 3 out of 9 (33.3%) were obese. Obesity was predominant in category A3 albuminuria, with nine patients (33.3%). Hypertension and diabetes emerge as predominant, regardless of the proteinuria category.

### 3.4. Outcomes

**Figure 5:**
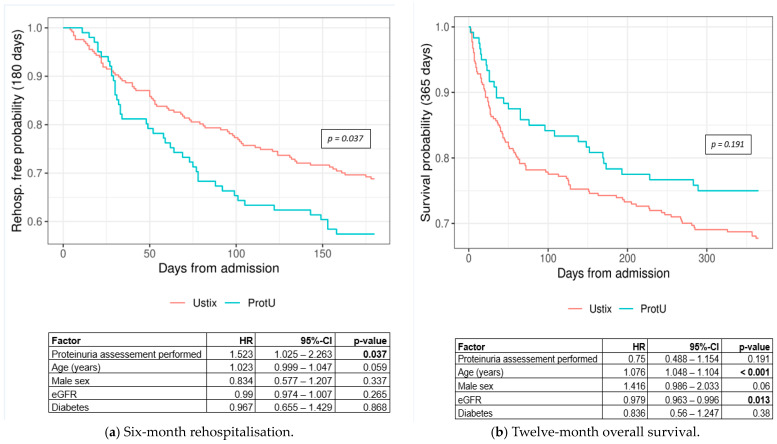
Kaplan–Meier curves for (**a**) 6-month rehospitalisation by group (log-rank *p*-value = 0.037) and (**b**) 12-month overall survival by diagnostic group (log-rank *p*-value = 0.191). Abbreviations: ProtU = UACR or UPCR testing available, Ustix = no assessment available, CI = confidence interval, eGFR = estimated glomerular filtration rate, HR = hazard ratio.

Cox-proportional hazards regression showed a significantly higher hazard for 6-month rehospitalisation in the ProtU group (hazard ratio (HR) = 1.523, 95% CI: 1.025–2.263, *p*-value = 0.037) (Figure 5a). Twelve-month overall survival did not differ significantly between the two groups (HR = 0.75, 95% CI: 0.488–1.154, *p*-value = 0.191) (Figure 5b). Numerically, mortality rates were higher in the Ustix group as opposed to the ProtU group.

**Figure 6:**
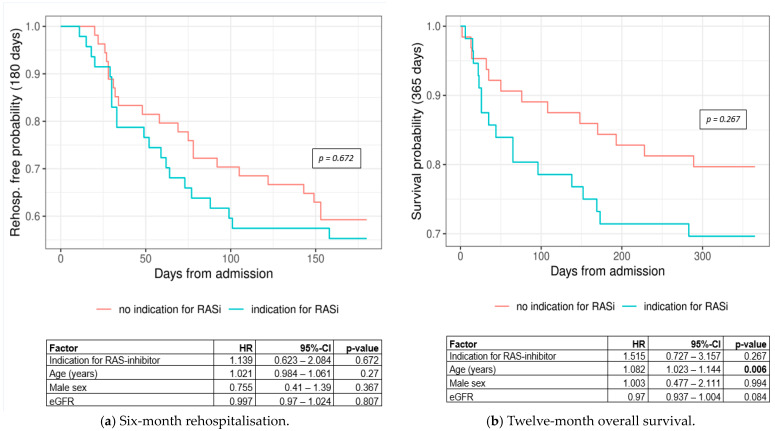
Kaplan–Meier curves for (**a**) 6-month rehospitalisation by treatment group (log-rank *p*-value = 0.672) and (**b**) 12-month overall survival by treatment group (log-rank *p*-value = 0.267). Abbreviations: CI = confidence interval, eGFR = estimated glomerular filtration rate, HR = hazard ratio, RASi = renin angiotensin system inhibitor.

Regarding the treatment indication, which correlates with the albuminuria category (cf. Table 4), the numbers for both rehospitalisation (HR = 1.139, 95% CI: 0.623–2.084, *p*-value = 0.672) (Figure 6a) and mortality (HR = 1.515, 95% CI: 0.727–3.157, *p*-value = 0.267) (Figure 6b) were not significantly different between the groups. Metrics show that individuals with an indication for RASi treatment had a worse outcome compared to those without an indication for treatment.

**Figure 7:**
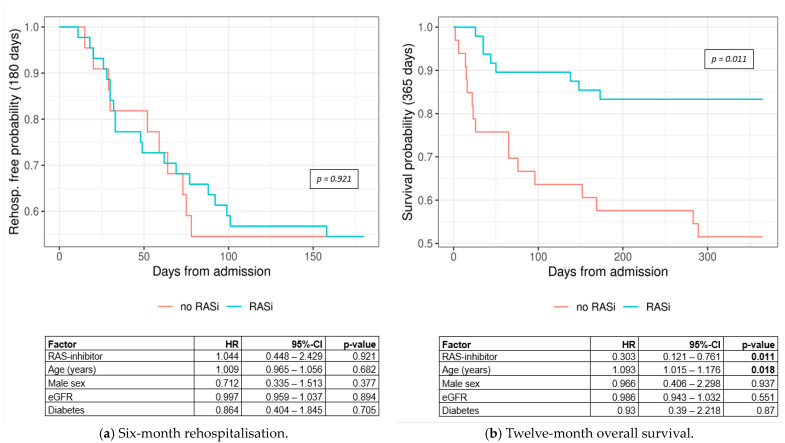
Kaplan–Meier curves for (**a**) 6-month rehospitalisation by treatment group (log-rank *p*-value = 0.921) and (**b**) 12-month overall survival by treatment group (log-rank *p*-value = 0.011). Abbreviations: CI = confidence interval; eGFR = estimated glomerular filtration rate; HR = hazard ratio; RASi = renin angiotensin system inhibitor.

In terms of adequate treatment, 6-month rehospitalisation did not differ significantly between the RASi and the no-RASi groups (HR = 1.044, 95% CI: 0.448–2.429, *p*-value = 0.921) (Figure 7a), though the numbers showed higher rehospitalisation rates for the no-RASi group. The 12-month survival was significantly better for patients receiving an antiproteinuric treatment (HR = 0.303, 95% CI: 0.121–0.761, *p*-value = 0.011) (Figure 7b). All Cox-proportional hazards regressions were adjusted for age, sex, eGFR, and diabetes.

## 4. Discussion

Our study on proteinuria management had three main findings. First, the compliance with the KDIGO 2012 guidelines for the assessment of proteinuria in CKD patients was low—a proteinuria measurement was seen to be available in almost 30% of the studied population. Secondly, a RASi was prescribed in only 59% of the cases for which an antiproteinuric treatment was indicated according to the guidelines described in Table 1. Third, in terms of 12-month survival, we found a better outcome in those who received RASi treatment.

The percentage of proteinuria assessment correlates with the numbers found in the literature so far, stating similar figures. A study conducted in 2011 by Allen et al. showed that proteinuria was assessed in only 30% of their cohort consisting of 11,774 patients with a category 3 or category 4 eGFR [27]. In 2017, a quality improvement programme for CKD was developed by Nitsch et al., stating that the ACR was measured in less than 30% of all patients [26]. In the Ustix group, half of those who had been assessed via a urinalysis should have obtained a protein quantification. It should be pointed out that it is most likely that these patients received a urinalysis primarily to rule out a urinary tract infection.

There is a myriad of reasons as to why no assessment may have been available in more than 70% of the cases. An important reason is the lack of awareness surrounding the fact that proteinuria has such a big impact on CKD progression [41]. This leads to physicians not knowing how to identify and/or manage pathological proteinuria [29].

In addition, older and more fragile patients may not live long enough to develop ESKD since the risk for all-cause mortality increases with age [42]. De Nicola et al. established a relationship between competing risks of ESKD and death without ESKD [11]. They plotted a figure showing that adults older than 65 years with an eGFR > 35 mL/min/1.73 m^2^ are more likely to die of other causes without ever acquiring ESKD [11]. Assessing proteinuria in geriatric patients with a low life expectancy is therefore not always appropriate, especially in category G3b—one might even say it would be ethically incorrect.

There appears to be a proportionality between the eGFR and the severity of the ACR. One would think that the lower the eGFR is, the more severe proteinuria should be (Table 3). However, this is not the case—ESKD goes along with reduced filtration, consequently mitigating albuminuria [20]. This could be a reasonable explanation for why the prevalence of severe albuminuria is higher in subjects with an eGFR of 15–29 in comparison to those with an eGFR < 15 mL/min/1.73 m^2^ (Table 3). We assume that individuals with severe albuminuria more often suffer from acute kidney failure during hospitalisations. In this context, nephrologists are consulted more often (Table 3) in order to help with the patient’s workup, which would therefore lead to a higher assessment rate [43].

In patients who had an indication for an antiproteinuric treatment, the prevalence was 33 out of 56 (59%), of which 12/17 (70.5%) with category A2 albuminuria were diabetic, which is coherent with other studies [26,29].

Because our study population is from 2022, it is important to note that there were no official guidelines at that time stating the below-mentioned recommendations. Although the KDIGO 2012 recommendations did not specifically mention SGLT2i or diuretics, there has been evidence suggesting that the latter can reduce proteinuria when combined with an ACEi or an ARB—all the more when associated with a low-protein diet [20,44,45]. In April 2024, an updated version of the KDIGO guidelines was released, in which major changes regarding proteinuria treatment were introduced.

There are numerous reasons why a suitable treatment was not prescribed for some patients. First, internists may assume that a quantification of proteinuria will not have a consequence in terms of treatment plans if the patient is already receiving RASi treatment due to hypertension. This could partly elucidate why some patients in the Ustix group (63.5%) received RASi treatment but were not tested for their UACR or UPCR. However, this is not quite the case, since higher doses of RASis could be given. The KDIGO 2024 version sates that a “RASi (ACEi or ARB) should be administered using the highest approved dose that is tolerated to achieve the benefits described because the proven benefits were achieved in trials using these doses” [37].

When the maximum dose of RASi is reached and significant residual proteinuria persists, diuretics of the mineralocorticoid receptor antagonist class, such as aldactone or thiazide diuretics, should be added to the treatment [20,46]. The KDIGO 2024 guidelines state the following: “We suggest a nonsteroidal mineralocorticoid receptor antagonist with proven kidney or cardiovascular benefit for adults with type 2 diabetes (T2D), an eGFR > 25 mL/min per 1.73 m^2^, normal serum potassium concentration, and albuminuria (>30 mg/g [>3 mg/mmol]) despite maximum tolerated dose of RAS inhibitor (RASi)” [37]. More recently, it has been established that SGLT2i can be administered as a nephroprotectant [20]. The KDIGO 2024 recommendations are stated as follows: “We recommend treating adults with CKD with an SGLT2i for the following: eGFR ≥ 20 mL/min per 1.73 m^2^ with urine ACR ≥ 200 mg/g (≥20 mg/mmol), or heart failure, irrespective of the level of albuminuria” [37].

Furthermore, as Nitsch et al. state, high doses of RASi or combination therapies can lower a patient’s BP, considerably causing hypotension and leading to falls or fainting, especially in elderly patients [26]. One could thus hypothesise that assessing proteinuria is not necessary, as treatment with RASi or its combinations will cause more damage to the patient than it benefits them. In addition to this, ethical and economic considerations may influence the decision not to assess proteinuria in the elderly population due to the lack of therapeutic consequences. Further, polypharmacy is known to promote falls in “*golden-agers*”, which may prompt hesitation amongst physicians to increase the dosage [47]. If there will not be a therapeutic consequence, assessing proteinuria is not necessary. Finally, age needs to be considered as well. O’Hare et al. evaluated the effect of RASi-centred treatment for ESKD in older patients [4,22]. The results showed that most patients fell in the groups with an NNT > 100, which suggests that there is a reduced marginal benefit regarding treatment with ACEis/ARBs to prevent ESKD [22]. In addition, there are controversies suggesting that the discontinuation of RASi treatment in advanced CKD does not lead to a clinically relevant change in the eGFR, and thus does not prevent ESKD [23,48].

We found that hypertension and diabetes were associated with dominant risk factors for proteinuria. Obese and diabetic patients were more likely to have received a proteinuria assessment and an appropriate treatment; this is coherent with other studies [26,28,29].

It is noteworthy that systolic and diastolic blood pressure levels were evaluated in the above-mentioned studies [26,29,49], as the guidelines suggest that patients with a permanent systolic BP > 130 mmHg or diastolic BP > 80 mmHg with moderate albuminuria should be treated with a RASi. However, hypertension should be assessed and treated in an ambulant setting. This is because during hospitalisations, numerous factors influence BP measurements. Factors such as pain, fatigue, anxiety, etc., are all causes for higher BP levels [50,51]. We opted not to take BP measurements into account, due to the fact that this is a retrospective study and thus the circumstances in which the patients’ BP was measured cannot be fully established.

In regard to rehospitalisation within 6 months, we found that the ProtU group (37.5%) had a significantly higher quota than the Ustix group (26.4%) (Table 2, Figure 5a). We hypothesise that these patients are more likely to have severe proteinuria and therefore would be more fragile and liable to diseases, leading to more hospitalisations [52]. Table 3 shows higher rehospitalisation rates for severe albuminuria (41.4%) compared to normal to mild albuminuria (35.5%) [53]. In addition, the risk of hospitalisation is higher with a low eGFR—the ProtU group had a median eGFR of 27 mL/min/1.73 m^2^, whereas that in the Ustix group was 34.2 mL/min/1.73 m^2^ [53,54,55].

In evaluating proteinuria 12-month survival using Cox-proportional hazards regression, the Ustix group showed a worse outcome, although the differences were not statistically significant (Table 2 and Figure 5b). We suggest that this is because proteinuria remained undetected, and therefore no corresponding treatment was initiated—resulting in worse outcomes [23]. In Table 3, we show that survival rates were lower in patients with moderate–severe albuminuria compared to mild albuminuria, which correlates with previous studies—14 (31.8%) vs. 4 (12.9%), respectively [56]. The current KDIGO guidelines suggest the following: “Continue ACEi or ARB in people with CKD even when the eGFR falls below 30 mL/min per 1.73 m^2^” [37].

Regarding the indication of RASi treatment, the group with an indication for treatment had more severe albuminuria (A3/ProtU ≥ 50 mg/mmol) and concomitant diabetes (A2); this therefore explains their higher rates of rehospitalisation (Figure 6a) and lower survival rates (Figure 6b). The group with a RASi indication had a 1.13-fold higher risk of rehospitalisation within six months. The group without an indication for a RASi had a 1.51-fold better chance of survival within one year. Despite the fact that our numbers were not statistically significant (probably due to the small sample size), we believe that there is an association between the severity of albuminuria and adverse outcomes.

A meta-analysis about the correlation between albuminuria and adverse outcomes confirms our findings [57]: in 114 cohorts, severe albuminuria was associated with a higher risk for all-cause mortality and hospitalisation. These findings emphasise the importance of albuminuria in assessing risk.

Figure 7a does not show an association between rehospitalisation and treatment with RASis. One cluster-randomised study conducted by Vazquez M. et al. compared patients with CKD, hypertension, and diabetes type 2 who either received guideline-based interventions or the usual care. Among the endpoints were death within 1 year and readmission within 30 days. Similar to our study, numerically, the intervention group did show a better outcome regarding hospitalisation compared to the group receiving usual care, but these numbers were not clinically relevant [58]. A larger trial would probably have shown an advantage regarding readmission [59].

When comparing patients with versus without RASi treatment, the outcomes of overall mortality were significantly better in the RASi group (Figure 7b) [60]. These results were to be expected due to the mechanism of action of the RASis on the kidneys—by reducing albuminuria and slowing the progression of CDK [61,62]. Our results are coherent with those of other studies, showing that the non-use/discontinuation of RASi treatment is associated with higher mortality [63,64].

Altogether, managing and slowing the progression of CKD is crucial—in 2017, 1.2 million people died from CKD, and it is expected that by 2040, this number will increase to 2.2–4 million deaths [6,7]. This highlights that risk factors such as proteinuria should be tackled from the onset, providing benefits to both patients and healthcare systems by preventing dialysis and transplantation [8]. A possible solution to improve proteinuria testing would be that for every eGFR < 60 mL/min/1.73 m^2^, a protein quantification is automatically performed in the laboratory on the next available urine specimen. Disseminating the relevant guidelines on the hospital’s homepage could make it easier and quicker for physicians to access the required information.

### Limitations

The retrospective design of this study comes with disadvantages. One downside is that the list of comorbidities in the discharge reports highly depends on the documentation performed by the attending clinicians. We did not evaluate BP due to the above-mentioned reasons. Another limitation is the number of patients that were excluded due to denied consent. Further, using a single-centre design can introduce biases that may impact the generalisability and validity of our findings. Due to the selection bias, our study does not represent a broader population.

Finally, as mentioned in the Materials and Methods section, we considered each case, instead of each patient, in our study. This meant that if one patient was hospitalised multiple times in 2022, each hospitalisation case would be included in the study—potentially leading to an artificial increase in the sample size and overrepresentation. Overall, 9.3% of patients were hospitalised more than once.

## 5. Conclusions

The results of this audit correlate with those of other studies, evidencing that the guidelines regarding assessing and managing proteinuria remain largely unmet and are not adhered to. Regular monitoring of proteinuria in CKD patients, as well as the initiation of RASi treatment when necessary, must be improved. We found that patients with diabetes are more likely to undergo proteinuria measurement, and treating proteinuria with a RASi translates into increased survival. Patients who had received adequate treatment with a RASi did not show lower hospitalisation rates than those who had received a corresponding treatment, and patients with an available proteinuria assessment did not show increased survival. Large prospective studies and/or post hoc studies are needed to confirm these findings.

## Figures and Tables

**Table 1:** KDIGO 2012 guidelines [1].

1. It is recommended that CKD is classified based on cause, GFR category, and albuminuria category.
2. It is recommended to assess GFR and albuminuria at least annually in people with CKD. Assess GFR and albuminuria more often for individuals at higher risk of progression, and/or where measurement will impact therapeutic decisions.
3. Confirm reagent strip positive albuminuria and proteinuria via quantitative laboratory measurements and express as a ratio to creatinine wherever possible, as follows:
3.1. urine albumin-to-creatinine ratio (ACR); or
3.2. urine protein-to-creatinine ratio (PCR).
4. An ARB or ACEi should be used in diabetic adults with CKD and urine albumin excretion ≥30–300 mg/24 h.
5. It is recommended that an ARB or ACEi is used in both diabetic and non-diabetic adults with CKD and urine albumin excretion >300 mg/24 h.
6. Refer to a specialist if there is a consistent finding of significant albuminuria (ACR ≥ 300 mg/g [≥30 mg/mmol] or AER ≥ 300 mg/24 h, approximately equivalent to PCR ≥ 500 mg/g [≥50 mg/mmol] or PER ≥ 500 mg/24 h).

Abbreviations: ACEi = angiotensin-converting enzyme inhibitor; ACR = albumin-to-creatinine ratio; AER = albumin excretion rate; ARB = angiotensin receptor blocker; CKD = chronic kidney disease; GFR = glomerular filtration rate; PCR = protein-to-creatinine ratio; PER = protein excretion rate.

**Table 2:** Baseline characteristics.

	Overall (n = 427)	ProtU Group (n = 120)	Ustix Group (n = 307)	*p*-Value
Gender (female), n (%)	196 (45.9)	45 (37.5)	151 (49.2)	
Age (years), median [IQR]	85 [79–89]	84 [78–88]	85 [79–89]	
**CKD staging**				<0.001 *
G3b (eGFR 44–30 mL/min/1.73 m^2^), n (%)	268 (62.8)	52 (43.3)	216 (70.4)	
G4 (eGFR 29–15 mL/min/1.73 m^2^), n (%)	123 (28.8)	47 (39.2)	76 (24.8)	
G5 (eGFR < 15 mL/min/1.73 m^2^), n (%)	36 (8.4)	21 (17.5)	15 (4.9)	
CKD-EPI eGFR, median [IQR]	32.8 [25–40]	27 [18–37]	34.2 [26–41]	<0.001 *^a^
**Comorbidities**				
Arterial hypertension, n (%)	326 (77.4)	87 (73.7)	239 (78.9)	0.297
Diabetes mellitus, n (%)	152 (36.1)	52 (44.1)	100 (33.0)	0.048 *
Cardiac diseases, n (%)	334 (79.3)	95 (80.5)	239 (78.9)	0.868
Respiratory diseases, n (%)	90 (21.4)	30 (25.4)	60 (19.8)	0.267
Obesity, n (%)	63 (15)	25 (21.2)	38 (12.5)	0.039 *
Dyslipidaemia, n (%)	136 (32.3)	38 (32.2)	98 (32.3)	1
History of smoking, n (%)	69 (16.4)	23 (19.5)	46 (15.2)	0.363
History of alcohol abuse, n (%)	8 (1.9)	3 (2.5)	5 (1.7)	0.692 ^b^
Acute infection, n (%)	28 (6.7)	51 (43.2)	116 (38.3)	0.431
Autoimmune disease, n (%)	28 (6.7)	6 (5.1)	22 (7.3)	0.552
Active malignancy, n (%)	42 (10.0)	13 (11.0)	29 (9.6)	0.718 ^b^
RASis, n (%)	261 (61.1)	66 (55)	195 (63.5)	0.13
Referral to nephrologist, n (%)	35 (8.2)	22 (18.3)	13 (4.2)	<0.001 *
Rehospitalisation within 6 months, n (%)	126 (29.5)	45 (37.5)	81 (26.4)	0.032 *
Death within 1 year, n (%)	132 (30.9)	31 (25.8)	101 (32.9)	0.192

Abbreviations: CKD-EPI = Chronic Kidney Disease Epidemiology Collaboration, eGFR = estimated glomerular filtration rate, IQR = interquartile range, ProtU = urine albumin-to-creatinine ratio or urine protein-to-creatinine ratio available, Ustix = no urine protein or albumin testing available, RASis = renin angiotensin system inhibitors, ^a^ Mann–Whitney U test, ^b^ Fisher’s Exact test, * *p* < 0.05.

**Table 3:** Assessing the urine albumin-to-creatinine ratio/urine protein-to-creatinine ratio in the ProtU group.

	Albuminuria Categories (UACR in mg/mmol)				Proteinuria (UPCR in mg/mmol)		
	Normal to Mild	Moderate	Severe	Missing UACR			Missing UPCR
	A1 < 3 n = 31	A2 3–30 n = 44	A3 > 30 n = 29	16	<50 n = 56	≥50 n = 31	33
G3b (eGFR 44–30 mL/min/1.73 m^2^), n (%)	16 (51.6)	22 (50)	7 (24.1)		24 (42.9)	10 (32.3)	
G4 (eGFR 29–15 mL/min/1.73 m^2^), n (%)	10 (32.3)	17 (38.6)	13 (44.8)		25 (44.6)	14 (45.2)	
G5 (eGFR < 15 mL/min/1.73 m^2^), n (%)	5 (16.1)	5 (11.4)	9 (31)		7 (12.5)	7 (22.6)	
RASis, n (%)	11 (35.5)	28 (63.6)	15 (51.7)		30 (53.6)	20 (64.5)	
Referral to nephron., n (%)	3 (9.7)	5 (11.4)	11 (37.9)		11 (19.6)	9 (29)	
Rehosp. 6 m, n (%)	11 (35.5)	14 (31.8)	12 (41.4)		19 (33.9)	16 (51.6)	
Death within 1 y, n (%)	4 (12.9)	14 (31.8)	9 (31)		17 (30.4)	9 (29)	

Abbreviations: eGFR = estimated glomerular filtration rate; nephron. = nephrologist; RASis = renin angiotensin system inhibitors; Rehosp. 6 m = rehospitalisation within 6 months; UACR = urine albumin-to-creatinine ratio; UPCR = urine protein-to-creatinine ratio; y = year.

**Table 4:** Indications of RASi treatments.

	Albuminuria Categories in (UACR in mg/mmol)		Proteinuria (UPCR in mg/mmol)	Overall RASi Prescriptions (%)
	Moderate w/ DT2—A2 3–30	Severe—A3 > 30	≥50	
Number of cases	17	29	31	56
RASis, n (%)	12 (70.5)	15 (51.7)	20 (64.5)	33 (59)

Abbreviations: DT2 = diabetes type 2, RASi = renin angiotensin system inhibitor, UACR = urine albumin-to-creatinine ratio, UPCR = urine protein-to-creatinine ratio, w/ = with.

## Data Availability

The data presented in this study can be made available from the corresponding author upon reasonable request. The data are not publicly available due to restrictions pertaining to data privacy.
